# Decreased Sclerostin Secretion in Humans and Mice With Nonalcoholic Fatty Liver Disease

**DOI:** 10.3389/fendo.2021.707505

**Published:** 2021-08-05

**Authors:** Fangli Zhou, Yan Wang, Yujue Li, Mengjia Tang, Shan Wan, Haoming Tian, Xiang Chen

**Affiliations:** ^1^Department of Endocrinology, Laboratory of Endocrinology and Metabolism, West China Hospital, Sichuan University, Chengdu, China; ^2^Department of Endocrinology, West China Hospital, Sichuan University, Chengdu, China

**Keywords:** sclerostin, NAFLD, fatty liver index, bone metabolism, triglyceride

## Abstract

**Objectives:**

Growing evidence argues for a relationship between liver and bone metabolisms. Sclerostin is a secreted glycoprotein and could antagonize osteoblast-mediated bone formation. Previous studies indicated that circulating sclerostin levels may be associated with metabolic parameters with inconsistent results. This study was designed to evaluate serum sclerostin in patients with or without nonalcoholic fatty liver disease (NAFLD) and to analyze its relationship with metabolic parameters in different populations.

**Methods:**

A cross-sectional study was designed and 168 NAFLD subjects and 85 control subjects were included in this study. Serum sclerostin and metabolic parameters were measured. Mouse models of NAFLD were also induced by high-fat diet. Bone structural parameters were determined using microCT and mRNA expression levels of sclerostin in bone and liver tissues were measured.

**Results:**

Our study suggested that circulating sclerostin levels were significantly lower in NAFLD subjects compared with normal controls. In NAFLD subjects, sclerostin was negatively correlated with multiple metabolic parameters, including waist circumference, urea, hepatic enzyme, gamma-glutamyl transpeptidase, and triglyceride, while such correlation was not significant in control subjects. Circulating sclerostin was also negatively correlated with fatty liver index in NAFLD subjects but not in control subjects. Mice fed on a high-fat diet had reduced bone mass and lower sclerostin expression levels in both the bone and liver tissues.

**Conclusions:**

Our study suggested that the liver-lipid-bone interactions may play a key role in the abnormal bone metabolism in NAFLD, and circulating sclerostin may be a surrogate marker to reflect bone metabolism status in NAFLD subjects.

## Introduction

Nonalcoholic fatty liver disease (NAFLD), which is closely associated with obesity, type 2 diabetes, and the metabolic syndrome, has become the most prevalent chronic liver disease today. In recent years, extrahepatic manifestations of NAFLD have attracted scientific attention (1, 2). Previous studies also indicated a possible liver - bone interaction (3). Low bone mineral density (BMD) and high risk of osteoporosis have been found in both pediatric and adult populations with NAFLD (4, 5). For example, Pardee et al. reported that obese children with NAFLD had significantly lower BMD Z-scores than obese children without NAFLD after controlling for age, sex, race, ethnicity, and total percent body fat (6). In Korean men, NAFLD has been demonstrated to be negatively associated with right-hip BMD and serum osteocalcin after adjusting for body mass index (BMI) and homeostasis model assessment of insulin resistance (HOMA-IR) in Korean men (7, 8). However, reverse results have also been reported. S. H. Lee et al. revealed a significantly negative association between the femoral neck (FN) BMD and NAFLD in men, while a positive correlation between lumbar spine BMD and NAFLD in postmenopausal women (7). It was suggested that fatty liver index (FLI), which is calculated using BMI, waist circumference (WC), serum triglyceride (TG), and gamma-glutamyltranspeptidase (γ-GGT) levels, was negatively associated with total hip, femoral neck, and whole-body BMD in Korean men, but not in women (9). A meta−analysis including studies in adults did not show a significant difference in BMD between patients with NAFLD and non-NAFLD (10), while meta-analysis including studies conducted in children or adolescents revealed significant differences in whole-body or lumbar BMD Z scores between children/adolescents with and without NAFLD (11). Therefore, the liver- bone interaction and the underlying mechanism deserve further investigations.

Sclerostin is a glycoprotein predominately secreted by osteocytes. Notably, although sclerostin could antagonize osteoblast-mediated bone formation through inhibiting the Wnt pathway, circulating sclerostin levels have been found to be positively associated with BMD in humans (12–14). It is speculated that circulating sclerostin may reflect numbers of osteocytes. Sclerostin has been found to be associated with metabolic abnormalities. Giuseppe Daniele et al. suggested that sclerostin levels were higher in impaired glucose regulation (IGR) subjects compared with normal glucose tolerant (NGT) individuals and are correlated with insulin resistance in skeletal muscle, liver, and adipose tissue (15). Higher sclerostin levels have also been found in type 2 diabetes (16, 17). NAFLD plays a key role in insulin resistance and type 2 diabetes. It is intriguing to explore whether sclerostin is correlated with NAFLD. S A. Polyzos et al. found a progressive decline in serum sclerostin levels from the controls (76.1 ± 6.8 pmol/L) to nonalcoholic simple steatosis (SS) (53.5 ± 6.4 pmol/L) and steatohepatitis (NASH) (46.0 ± 8.1 pmol/L) patients (*p* = 0.009) (3). Our clinical study also demonstrated that circulating sclerostin levels were significantly lower in NAFLD subjects than normal controls and were significantly correlated with multiple metabolic parameters. Although sclerostin is predominately expressed by osteocytes, sclerostin mRNA was also detected in liver tissues in human and mice. Therefore, we also built mouse models of NAFLD using a high-fat diet (HFD) to compare the mRNA expression levels of sclerostin in both the bone and liver tissues between mice fed on a control diet (CON) or HFD.

## Materials and Methods

### Subjects

This study was approved by the Ethics Committee of the West China Hospital. All participants gave written informed consent. NAFLD group included patients with ultrasound found fatty liver, and there were no causes for secondary hepatic fat accumulation due to significant alcohol consumption, malnutrition, hepatitis B virus, and hepatitis C virus. Exclusion criteria include: (1). Patients with alcohol consumption more than 140g/week for males and 70g/week for females; (2). There was a past history of hepatitis B virus and hepatitis C virus; (3). Ultrasound showed hepatomegaly; (4). BMI<16 and incomplete data. 168 NAFLD patients were included in our study. 85 age- matched control subjects without ultrasound found fatty liver were recruited from West China hospital’s physical examination center. None of the participants were on any medications known to cause hepatic steatosis or taking vitamin supplements.

### Anthropometric Measurements

Anthropometric measurements were performed for all participants and were recorded by trained staff. Body height, weight, blood pressure, and WC were recorded. BMI was calculated as weight (kg) divided by height (m^2^) squared. Measurements were carried out twice by two independent interviewers.

### Laboratory Assessments

Venous blood samples were collected to measure lipids, liver function, and other biochemical parameters after fasting for ≥ 8 h. Total cholesterol (TC), TG, low-density lipoprotein cholesterol (LDL-C), high-density lipoprotein cholesterol (HDL-C), alanine transaminase (ALT), aspartate aminotransferase (AST), γ-GGT, urea, alkaline phosphatase (ALP), ferritin, and alpha-fetoprotein (AFP) were assessed in all the patients using automated, standardized equipment from the Clinical Laboratory of West China Hospital. Plasma glucose levels were tested using a hexokinase enzymatic technique. Serum insulin was measured using a radioimmunoassay (Beijing North Institute of Biological Technology). Circulating sclerostin levels were measured using an ELISA kit from Abcam (ab221836, Cambridge, UK). The detection limit of the assay is 6 pg/mL with a range of 31.1 – 2000 pg/mL. The intra- and inter-assay precisions are 4.8 and 8.6%, respectively. HOMA-IR was used to measure the insulin resistance as the equation: HOMA-IR= [fasting plasma glucose (mmol/L) ×fasting insulin (pmol/L)]/22.5. Fatty liver index (FLI), calculated from serum TG, BMI, WC, and γ-GGT, has been used as a surrogate marker of NAFLD and a screening test in epidemiologic studies. FLI was calculated by the following formula: FLI = [e^0.953 × ln (TG) + 0.139 × BMI + 0.718 × ln (GGT) + 0.053 × WC - 15.745^/(1 + ^e0.953 × ln (TG) + 0.139 × BMI + 0.718 × ln (GGT) + 0.053 × WC - 15.745^)] × 100 (18).

### Abdominal Ultrasonography

A high-resolution B-mode ultrasound probe (IU22, Philips, Netherlands) equipped with a 7.5 MHz linear array was used to measure the fatty liver. Participants were asked to maintain in the supine position with the right arm raised above the head during the examination. The liver’s fatty infiltration was diagnosed by two experienced sonographers unaware of the study’s aims and blinded to the laboratory results. A fatty liver was defined as the presence of hyperechogenic liver parenchyma compared to the kidney or spleen parenchyma.

### Animals and Interventions

Male C57BL/6 mice were maintained in the temperature- and light-controlled pathogen-free barrier facility under a 12-h light–12-h dark cycle and had free access to water and diet. At the age of 8 weeks, mice were divided into 2 groups with 6 animals each either on a standard chow diet (64% carbohydrate, 10% fat, and 26% protein) or a high-fat diet (28% carbohydrate, 60% fat, and 12% protein) for 12 weeks.

At the end of the experiment, mice were euthanized with an intraperitoneal injection of sodium pentobarbital. Bone and liver tissues were fixed in 4% formalin, embedded in paraffin, and stained with Hematoxylin and Eosin for histopathological analysis. Oil Red O staining was also performed in the frozen liver sections. The left femora were immersed into 4% paraformaldehyde immediately for measurement of bone structural parameters.

Bone structural parameters of mice, including trabecular bone volume (Tb. BV/TV), cortical bone volume (Cort. BV/TV), trabecular number (Tb·N), trabecular thickness (Tb·Th), and trabecular separation (Tb·Sp), were measured using microcomputed tomography (μCT) (MicroCT80, Scanco Medical AG, Bassersdorf, Switzerland), as previously described (19).

Total RNA was extracted from femur distal metaphyses (which was devoid of bone marrow) and liver tissues using Trizol reagent according to the manufacturer’s protocol (Invitrogen, Frederick, USA). 1 μg RNA was reversely transcribed into cDNA with PrimeScript™ RT reagent kit (TaKaRa Biotechnology Co., Ltd., Dalian, China). Following reverse transcription, the cDNA (2 μl) was amplified and quantified (Bio-Rad laboratories, Inc., California, USA). The sequence of oligonucleotide primers was listed in the following: sclerostin forward primer: CCTCATCTGCCTACTTGTGC (5′–3′); sclerostin reverse primer: GGTCTGGTTGTTCTCAGGAGG (5′–3′). Relative gene expression levels were normalized to beta-actin and analyzed with the 2^-ΔΔCt^ method.

### Statistical Analysis

Data were analyzed using SPSS v. 16.0 software. Shapiro–Wilk’s test was used to verify the normal distribution of continuous data before each analysis. Analysis showed that HOMA-IR, sclerostin, ALT, TG, γ-GGT, ferritin and FLI were not distributed normally. Data are presented as mean ± standard deviation (SD) for normally distributed data. Otherwise, non-normally distributed data were presented as median (quartiles) and were transformed, using the natural logarithms, before each analysis. The significance of group differences was evaluated using independent samples t-Test for continuous variables, while Chi-square test was performed for categorical variables. Pearson’s correlation test or Spearman’s correlation test was used to determine the correlation between sclerostin levels with various parameters. Partial correlation was used to eliminate the influence of potential confounding factors. The statistical significance was set at p<0.05 (two-tailed).

## Results

### Clinical Study

As expected, the metabolic parameters were significantly different between NAFLD subjects and normal controls (**Table 1**). NAFLD subjects had significantly higher BMI, WC, Waist-to-Hip ratio (WHR), blood pressure levels, urea, AST, ALT, fasting blood glucose levels, HOMA-IR, TC, TG, LDL-C, γ-GGT, ALP, and ferritin levels, as well as significantly lower HDL-C levels, compared with normal controls. Height, fasting insulin, and AFP levels were not different between the two groups. Circulating sclerostin levels were significantly lower in NAFLD subjects than normal controls.

**Table 1:** Baseline characteristics and biochemical indices.

	Control (n=85)	NFALD (n=168)	*p* values
Age (years)	43.27 ± 11.19	47.74 ± 10.35	0.002
Gender (male/female)	48/37	119/49	0.000
Body weight (kg)	61.48 ± 9.50	70.73 ± 10.53	0.000
Height (cm)	163.27 ± 7.97	165.08 ± 8.47	0.111
BMI (kg/m^2^)	22.99 ± 2.67	25.86 ± 2.48	0.000
Waist circumference (cm)	77.96 ± 7.56	88.19 ± 7.60	0.000
WHR	0.83 ± 0.06	0.91 ± 0.05	0.000
SBP (mmHg)	116.16 ± 16.36	121.75 ± 16.75	0.014
DBP (mmHg)	71.60 ± 10.06	77.54 ± 10.44	0.000
Urea (umol/L)	320.31 ± 82.03	399.57 ± 96.82	0.000
ALT (IU/L)	19.00(14.00-26.00)	38.00(24.00-56.75)	0.000
AST (IU/L)	23.00 ± 9.15	32.27 ± 15.48	0.000
Fasting plasma glucose (mmol/L)	5.14 ± 0.52	5.87 ± 1.84	0.000
Fasting insulin (pmol/L)	14.04 ± 6.77	15.35 ± 7.56	0.163
HOMA-IR	3.08(2.02-4.50)	3.62(2.36-5.22)	0.013
TC (mmol/L)	4.76 ± 0.74	5.17 ± 0.99	0.001
TG (mmol/L)	1.16 (0.90-1.62)	2.13(1.57-3.04)	0.001
LDL-C (mmol/L)	2.57 ± 0.64	2.80 ± 0.78	0.018
HDL-C (mmol/L)	1.59 ± 0.41	1.27 ± 0.30	0.000
γ-GGT (IU/L)	16.00 (12.00-23.00)	35.50(25.00-66.75)	0.000
ALP (IU/L)	67.18 ± 18.43	78.38 ± 21.39	0.000
Ferritin (ng/mL)	111.23 (53.27-152.84)	209.18(125.38-303.32)	0.000
AFP (ng/mL)	3.75 ± 2.13	3.52 ± 1.36	0.320
Sclerostin (pg/mL)	462.60(346.95-617.15)	362.25(189.78-489.40)	0.000
FLI	12.42 (6.19-23.20)	52.77 (34.63-70.47)	0.000

NAFLD, nonalcoholic fatty liver disease; BMI, body mass index; WC, waist circumference; WHR, Waist-to-Hip ratio; SBP, systolic blood pressure; DBP, diastolic blood pressure; ALT, alanine aminotransferase; AST, aspartate aminotransferase; HOMA-IR, homeostasis model assessment of insulin resistance; TC, total cholesterol; TG, triglyceride; LDL-C, low density lipoprotein; HDL-C, high-density lipoprotein; γ-GGT, gamma-glutamyltranspeptidase; ALP, alkaline phosphatase; AFP, alpha fetoprotein; FLI, fatty liver index.

For normal controls, circulating sclerostin was found to be positively associated with age, fasting insulin levels, and HOMA-IR and negatively correlated with HDL-C levels through Pearson analysis (**Table 2**). After adjusting for age and WC, these correlations were still significant (**Table 3**). For NAFLD subjects, sclerostin showed a positive correlation with age and a negative correlation with height, body weight, WC, DBP, urea, ALT, AST, fasting insulin levels, HOMA-IR, TC, TG, γ-GGT, and ALP levels by Pearson analysis (**Table 2**). After adjustment for age and WC, sclerostin was still negatively correlated with DBP, ALT, AST, HOMA-IR, TC, TG, γ-GGT, ALP and FLI (**Table 3**). Further analysis showed that the correlation between sclerostin with BW, BMI, WC, WHR, urea, insulin, HOMA-IR, TG and HDL-C are different between two groups (**Table 2**). It seems that sclerostin are closely associated with TG, BW and BW-related parameters in NAFLD subjects, while are closely correlated with insulin, HOMA-IR and HDL-C in controls (**Table 2**).

**Table 2:** Correlation between sclerostin and other parameters.

Parameters	Control		NFALD		Comparence of correlation		
	*r*	*p*	*r*	*p*	95% confidence interval		*p*
Age (years)	0.343	0.001	0.233	0.002	-0.136	0.340	0.374
Gender	-0.115	0.297	0.102	0.189	-0.470	0.047	0.107
Body weight (kg)	0.165	0.141	-0.224	0.004	0.128	0.632	0.004
Height (cm)	0.046	0.682	-0.183	0.020	-0.033	0.483	0.087
BMI (kg/m^2^)	0.176	0.116	-0.128	0.104	0.041	0.551	0.023
Waist circumference (cm)	0.185	0.099	-0.183	0.020	0.106	0.612	0.006
WHR	0.175	0.117	-0.126	0.111	0.038	0.549	0.025
SBP (mmHg)	-0.056	0.582	-0.047	0.551	-0.268	0.253	0.947
DBP (mmHg)	-0.062	0.099	-0.236	0.002	-0.083	0.430	0.187
Urea	0.160	0.143	-0.176	0.022	0.073	0.582	0.012
ALT (U/L)	-0.034	0.755	-0.216	0.005	-0.077	0.438	0.170
AST (U/L)	-0.129	0.238	-0.217	0.005	-0.165	0.345	0.512
Fasting plasma glucose (mmol/L)	0.111	0.312	-0.055	0.480	-0.098	0.420	0.218
Fasting insulin (pmol/L)	0.217	0.047	-0.174	0.024	0.130	0.632	0.003
HOMA-IR	0.243	0.025	-0.161	0.037	0.144	0.643	0.002
TC (mmol/L)	-0.029	0.793	-0.173	0.025	-0.116	0.402	0.281
TG (mmol/L)	0.178	0.103	-0.256	0.001	0.174	0.675	0.001
LDL-C (mmol/L)	0.061	0.579	-0.121	0.118	-0.081	0.438	0.176
HDL-C (mmol/L)	-0.256	0.018	0.151	0.051	-0.645	-0.148	0.002
γ-GGT (U/L)	-0.067	0.541	-0.280	0.000	-0.042	0.467	0.103
ALP (μg/L)	-0.076	0.489	-0.165	0.033	-0.169	0.348	0.504
Ferritin (ng/mL)	0.051	0.645	-0.136	0.079	-0.076	0.443	0.164
AFP (ng/mL)	-0.096	0.381	0.013	0.868	-0.365	0.155	0.419

NAFLD, nonalcoholic fatty liver disease; BMI, body mass index; WC, waist circumference; WHR, Waist-to-Hip ratio; SBP, systolic blood pressure; DBP, diastolic blood pressure; ALT, alanine aminotransferase; AST, aspartate aminotransferase; HOMA-IR, homeostasis model assessment of insulin resistance; TC, total cholesterol; TG, triglyceride; LDL-C, low density lipoprotein; HDL-C, high-density lipoprotein; γ-GGT, gamma-glutamyltranspeptidase; ALP, alkaline phosphatase; AFP, alpha fetoprotein.

**Table 3:** Correlation between sclerostin and other parameters after adjustment of WC and age.

Parameters	Control		NFALD	
	*r*	*p*	*r*	*p*
DBP (mmHg)	-0.216	0.058	-0.170	0.033
AST (U/L)	-0.123	0.284	-0.174	0.029
ALT (U/L)	-0.023	0.841	-0.200	0.012
Fasting insulin (pmol/L)	0.270	0.018	-0.130	0.104
HOMA-IR	0.269	0.017	-0.159	0.046
TC (mmol/L)	-0.070	0.545	-0.189	0.018
TG (mmol/L)	-0.100	0.384	-0.244	0.002
HDL-C (mmol/L)	-0.233	0.040	-0.112	0.164
γ-GGT (U/L)	-0.116	0.311	-0.186	0.020
ALP (μg/L)	-0.179	0.116	-0.165	0.039
FLI	0.025	0.830	-0.161	0.045

WC, waist circumference; NAFLD, nonalcoholic fatty liver disease; DBP, diastolic blood pressure; AST, aspartate aminotransferase; ALT, alanine aminotransferase; HOMA-IR, homeostasis model assessment of insulin resistance; TC, total cholesterol; TG, triglyceride; HDL-C, high-density lipoprotein; γ-GGT, gamma-glutamyltranspeptidase; ALP, alkaline phosphatase; FLI, fatty liver index.

**Figure 1:**
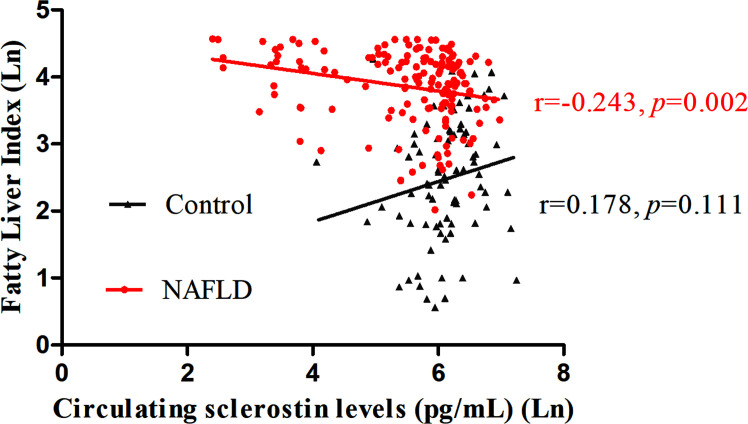
Circulating sclerostin was negatively correlated with FLI in NAFLD subjects (*r*=-0.243, *p*=0.002), but not in control subjects (*r*=0.178, *p*=0.111). FLI, fatty liver index; NAFLD, nonalcoholic fatty liver disease.

FLI serves as a surrogate marker for liver fat content. Our study revealed a negative correlation between circulating sclerostin with FLI in NAFLD subjects (*r*=-0.243, *p*=0.002), while no correlation was found in normal control (*r*=0.178, *p*=0.111) (**Figure 1**). Further analysis showed that the correlation of NAFLD and controls are statistically different (95% confidence interval: -0.663, -0.160; *p*=0.002). After adjustment for age and WC, the correlation was still significant in NAFLD subjects (**Table 3**).

### Animal Study

**Figure 2:**
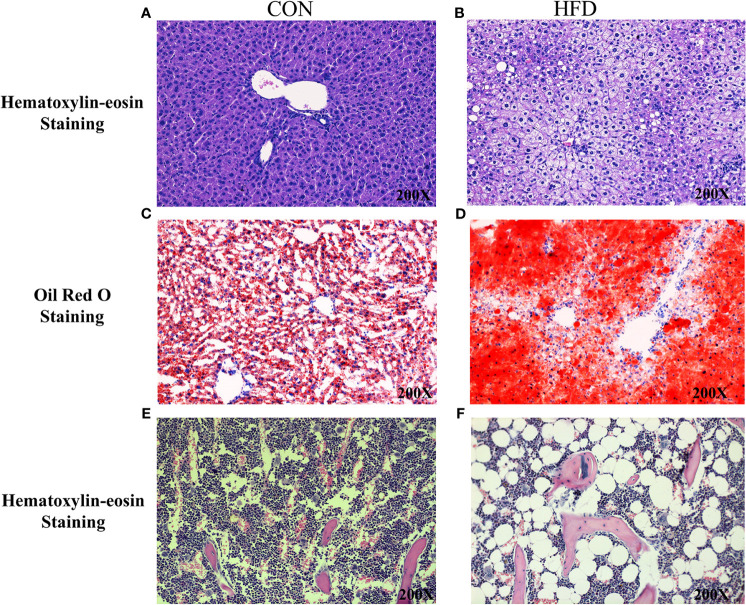
Histological analyses of fat deposition in liver and bone marrow of mice. **(A, B)** Hematoxylin and Eosin staining showed more lipid droplets accumulated in the liver tissues of HFD-fed mice, compared with mice fed on control diets. **(C, D)** Oil Red O staining showed more lipid droplets accumulated in the liver tissues of HFD-fed mice. **(E, F)** Hematoxylin and Eosin staining revealed more fat vacuoles in the bone marrow of mice fed on HFD. CON, control; HFD, high-fat diet.

**Figure 3:**
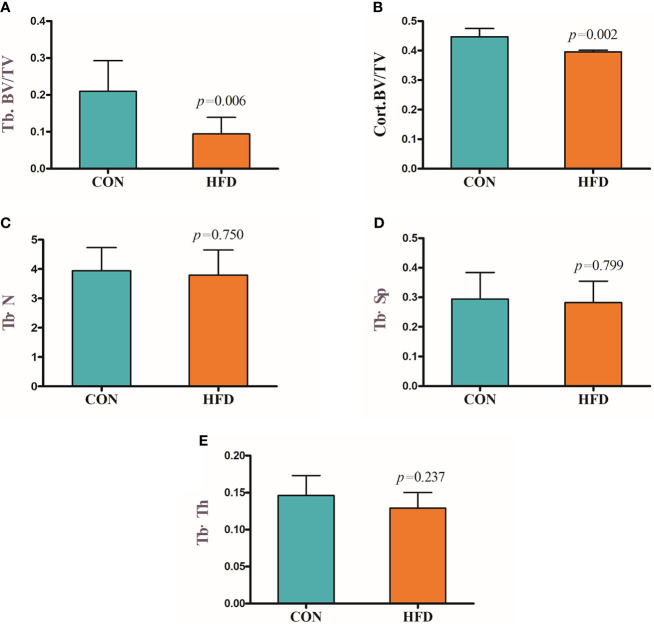
Decreased bone mass in mice fed on a high-fat diet. **(A)** Trabecular bone volume (Tb. BV/TV). **(B)** Cortical bone volume (Cort. BV/TV). **(C)** Trabecular bone number (Tb. N). **(D)** Trabecular separation (Tb·Sp). **(E)** Trabecular bone thickness (Tb·Th). CON, control; HFD, high-fat diet.

**Figure 4:**
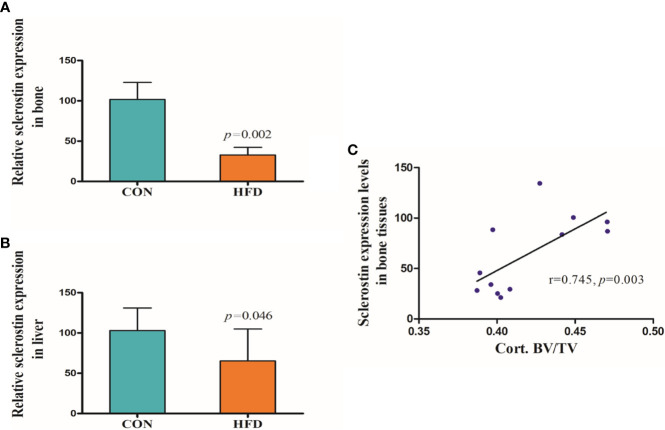
Expression levels of sclerostin. **(A)** Sclerostin expression was significantly decreased in bone tissues of HFD-fed mice (*p*=0.002). **(B)** Sclerostin expression was significantly decreased in liver tissues of HFD-fed mice (*p*=0.046). **(C)** Sclerostin expression levels in bone tissues were positively correlated with Cort. BV/TV (*r*=0.745, *p*=0.003). CON, control; HFD, high-fat diet.

Hematoxylin and Eosin and Oil Red O staining showed more lipid droplets accumulated in the liver tissues of HFD-fed mice compared with mice fed on a control diet (**Figures 2A–D**). More fat vacuoles were found in the bone marrow of mice fed on a high-fat diet (**Figures 2E, F**). Tb. BV/TV and Cort. BV/TV were significantly decreased in mice fed on a high-fat diet compared to mice on a control diet, while Tb. N, Tb. Sp and Tb. Th were not significantly different between groups (**Figure 3**). The mRNA expression levels of sclerostin were significantly lower in both the bone and liver tissues of HFD-fed mice than those of mice on a control diet (**Figures 4A, B**). Sclerostin expression levels in bone tissues were positively correlated with Cort. BV/TV (**Figure 4C**).

## Discussion

Our study suggested that circulating sclerostin levels were significantly lower in NAFLD subjects compared with normal controls, which was consistent with a previous study (3). Our study also indicated that sclerostin was correlated with multiple metabolic parameters, especially WC, hepatic enzyme, γ-GGT, and TG. Although sclerostin is predominantly expressed by osteocytes, sclerostin mRNA has been detected in other human and mouse tissues, including cartilage, liver, kidney, and heart (20). Research conducted by M E. Brunkow et al. suggested that sclerostin expression levels in human and mouse liver were only lower than those of bone and cartilage (21). Therefore, we also conducted an animal study to compare the expression levels of sclerostin in bone and liver tissues of mice fed on either a control diet or HFD. Mice fed on HFD showed significantly lower sclerostin expression levels in both the bone and liver tissues, especially the bone tissues. Since the mRNA expression levels of sclerostin in liver tissues were extraordinarily low (data are not shown), when compared with the expression levels of sclerostin in bone tissues, it was speculated that the decreased sclerostin levels in NAFLD subjects may mainly reflect reduced sclerostin secretion from bone tissues.

Previous studies suggested that sclerostin expression is regulated by several factors, including age, estrogen, parathyroid hormone (PTH), and mechanical loading et al. (22). Our study also found a significantly positive correlation between sclerostin and age. Our study indicated that the correlation between sclerostin with metabolic parameters are different between two groups. In NAFLD subjects, sclerostin was closely and negatively correlated with TG, BW and BW-related indicators, while in controls, sclerostin was positively and significantly correlated with insulin and HOMA-IR, and negatively correlated with HDL-C. However, in subjects with NFALD, sclerostin was negatively associated with insulin and HOMA-IR. It seems that body weight gain and accompanying metabolic abnormalities may play a key role in the reduced sclerostin levels in NAFLD subjects. In this study, the sex distribution is significantly different between groups. However, our study indicated that gender may not play a role in the different sclerostin levels between two groups. In both the NAFLD patients and controls, circulating sclerostin levels were not different between male and female (**Supplementary Figure 1**), and no significant correlation between sclerostin and gender was found (*r*=-0.115, *p*=0.297 in NAFLD; *r*=0.102, *p*=0.189 in controls). WC, γ-GGT, and TG are also key risk factors of NAFLD and are used to calculate FLI. In this study, we also analyzed the possible association between sclerostin and FLI in different populations. Similarly, circulating sclerostin was significantly and negatively correlated with FLI in NAFLD subjects, while it was not associated with FLI in control subjects. Our study may suggest that in normal subjects without NAFLD, these metabolic indexes showed no obvious influence on sclerostin secretion, while in NAFLD subjects, these multiple metabolic abnormalities may inhibit sclerostin secretion mediated by a direct or indirect mechanism. The more obvious the metabolic abnormalities were, the more prominent the inhibitory effects on sclerostin expression.

NAFLD, defined by the presence of hepatic steatosis in the absence of other causes for hepatic fat accumulation, is actually a multisystemic clinical disease with extrahepatic manifestations including cardiovascular disease, type 2 diabetes, chronic kidney disease, hypothyroidism, polycystic ovarian syndrome, and psoriasis (1). Decreased BMD and increased risks of osteoporotic fractures have been observed in NAFLD subjects (5), and the underlying mechanism linking fat accumulation in the liver with abnormal bone metabolism has attracted increasing interest recently (5). Chronic inflammation, vitamin D deficiency, growth hormone (GH)/insulin-like growth factor 1 (IGF-1) axis, insulin resistance, limited physical activity, some adipokines like adiponectin and leptin, as well as marrow adipose tissue (MAT) have been proposed as possible mediators of mutual interactions among the skeleton, fatty tissue, and liver (23).

The liver plays a key role in lipid, glucose, and energy metabolism. NAFLD reflects a local manifestation of systemic metabolic abnormalities. These abnormities are closely associated with increased body fat content and abnormal lipid metabolism. Our previous and current studies have found a decreased bone mass and increased adipocytes in the bone marrow of mice fed on HFD, indicating MAT may be critically involved in the interplay between bone and liver (19). MAT, which is functionally distinct from both white and brown adipose, can contribute to systemic and skeletal metabolism (23). The previous study found no correlation between MAT with any measure of metabolic risk, including WHR, blood pressure, carotid intima-media thickness, insulin resistance, or lipid profile in young, healthy individuals, while such association was noticed in obese women as well as in patients with type 2 diabetes, indicating MAT may be activated by these metabolic abnormalities. Our present study also revealed similar phenomena. A significant and negative correlation was observed between sclerostin with multiple metabolic indexes, including FLI, in NAFLD subjects, while such correlation was not significant in control subjects without NAFLD. Sclerostin acts as a negative regulator of bone formation by inhibiting the Wnt pathway, while most previous studies revealed a positive correlation between circulating sclerostin levels and BMD in human subjects (24). Our animal study also found that sclerostin expression levels in bone tissues were positively correlated with Cort. BV/TV. Therefore, the decreased circulating sclerostin levels in NAFLD patients may reflect the reduced bone mass and abnormal bone metabolism in these subjects to some degree. MAT may be involved in this process, and the underlying mechanism needs further investigation.

It has been suggested that sclerostin could directly increase adipogenesis in mouse pre-adipocytes and enhance adipocyte differentiation in 3T3-L1 cells (25, 26). However, in our current study, we showed that mice on HFD had more adipocytes in bone marrow while lower sclerostin expression in bone tissues. The inhibited sclerostin expression may reflect lower bone mass with decreased osteocyte numbers in these mice on HFD. However, there may be an interaction between sclerostin and MAT, and enhanced MAT may show some regulatory effects on sclerostin expression. The relevant studies are under way.

Previous studies found increased sclerostin levels in type 2 diabetic patients compared with age-matched controls and type 1 diabetic patients, and sclerostin increased with the number of metabolic syndrome features (16, 17). In post-menopausal type 2 diabetic women with NAFLD and significant fibrosis, sclerostin levels were also higher than those of type 2 diabetic patients without NAFLD (27). In our study, a positive association between sclerostin levels with insulin and HOMA-IR was found in control subjects without NAFLD, while a negative correlation was found between sclerostin and HOMA-IR in NAFLD subjects. Furthermore, NAFLD patients showed a significant correlation between sclerostin levels with lipid profiles, especially TG levels, even after adjusting for confounding factors. These discrepancies may suggest that pathways involved in bone metabolism are different between T2DM and NAFLD.

Our study has certain limitations. First, the clinical study was a cross-sectional study, and thus no causal relationship could be established. Second, the liver biopsy was not performed due to possible risks for participants. Furthermore, BMD and bone turnover markers, including bone-specific alkaline phosphatase (B-ALP), procollagen I N-terminal propeptide (PINP), and cross-linked type I collagen (CTx), have not been measured in the clinical study. However, the animal experiment was performed and showed from different aspects that sclerostin expression in both the bone and liver tissues was indeed decreased in mice with NAFLD induced by HFD. Our animal study also revealed a positive correlation between sclerostin expression levels in bone tissues and Cort. BV/TV.

## Conclusions

Our study suggested that circulating sclerostin levels were significantly decreased in NAFLD subjects and were negatively correlated with multiple metabolic parameters, including FLI. Mice with NAFLD induced by HFD showed decreased bone mass and lower sclerostin expression in bone and liver tissues. Our study indicated that the liver-lipid-bone interactions may play a key role in the abnormal bone metabolism in NAFLD.

## Data Availability Statement

The original contributions presented in the study are included in the article/**Supplementary Material**. Further inquiries can be directed to the corresponding author.

## Ethics Statement

The studies involving human participants were reviewed and approved by the Ethics Committee of the West China Hospital. The patients/participants provided their written informed consent to participate in this study. The animal study was reviewed and approved by the Ethics Committee of the West China Hospital.

## Author Contributions

FZ and XC designed research, collected data, and wrote the manuscript. YW, YL, MT, and SW performed animal study and collected clinical data. HT designed the study and revised paper. All authors contributed to the article and approved the submitted version.

## Funding

The study was supported by West China Hospital, Sichuan University (Grant Nos. ZYGD18022 and 2020HXBH028 to FZ), China Postdoctoral Science Foundation (Grant No. 2020M670060ZX to FZ) and West China Hospital, Sichuan University (Grant No 2018HXFH009 to FZ). This work was also supported by the 1.3.5 project for disciplines of excellence, West China Hospital, Sichuan University (No. ZYGD18022 to HT).

## Conflict of Interest

The authors declare that the research was conducted in the absence of any commercial or financial relationships that could be construed as a potential conflict of interest.

## Publisher’s Note

All claims expressed in this article are solely those of the authors and do not necessarily represent those of their affiliated organizations, or those of the publisher, the editors and the reviewers. Any product that may be evaluated in this article, or claim that may be made by its manufacturer, is not guaranteed or endorsed by the publisher.

## Supplementary Material

Supplementary Figure 1 Comparence of circulating sclerostin levels between male and female. **(A)** Controls. **(B)** NAFLD subjects.
