# Supplementary figures and images for: Decreased Sclerostin Secretion in Humans and Mice With Nonalcoholic Fatty Liver Disease
Source: Front Endocrinol (Lausanne). 2021 Aug 5;12:707505. doi: 10.3389/fendo.2021.707505 (PMC8374147; doi:10.3389/fendo.2021.707505)

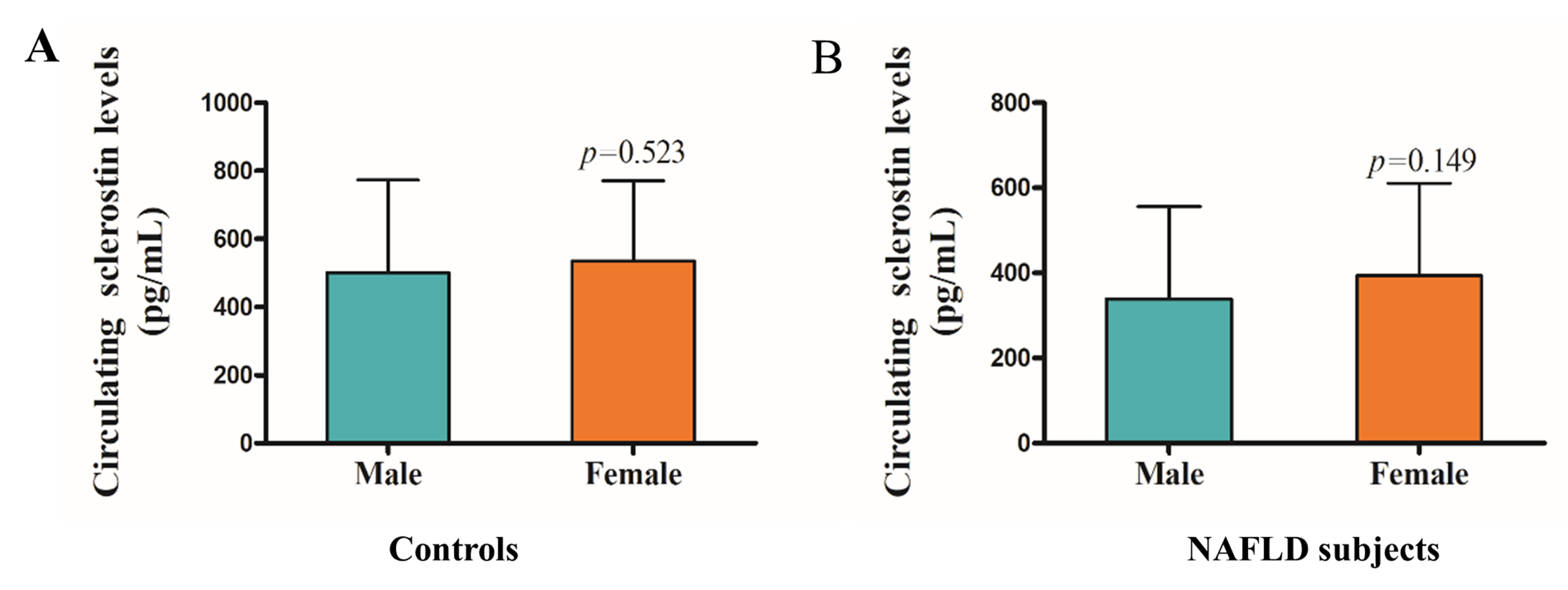

Supplement: Supplementary Figure 1 — Comparence of circulating sclerostin levels between male and female. (A) Controls. (B) NAFLD subjects. [file Image_1.tif]
